# 2-*n*-Butyl-1,2-benzisothia­zol-3(2*H*)-one 1,1-dioxide

**DOI:** 10.1107/S1600536808007733

**Published:** 2008-04-04

**Authors:** Guan-Ping Yu, Zhong-Jie Xu, Liang-Zhong Xu, Haji Akber Aisa

**Affiliations:** a Xinjiang Technical Institute of Physics and Chemistry, Chinese Academy of Science, Urumqi, 830011, People’s Republic of China; b Graduate School of the Chinese Academy of Science, Beijing, 100039, People’s Republic of China; c College of Chemistry and Molecular Engineering, Qingdao University of Science and Technology, Qingdao, 266042, People’s Republic of China

## Abstract

The crystal packing of the title compound, C_11_H_13_NO_3_S, exhibits weak inter­molecular C—H⋯O hydrogen bonding, which links mol­ecules related by translation along the *b* axis into chains, and π–π inter­actions [centroid–centroid distance of 3.778 (2) Å between benzene rings].

## Related literature

For similar crystal structures, see: Feeder & Jones (1994 ▶, 1996 ▶); Glidewell *et al.* (2000 ▶). For related literature, see: Xiong (2004 ▶); Rice & Pettit (1954 ▶).
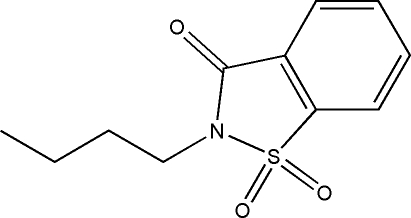


## Experimental

### 

#### Crystal data

C_11_H_13_NO_3_S
*M* *_r_* = 239.28
Triclinic, 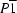

*a* = 7.3130 (15) Å
*b* = 7.7219 (15) Å
*c* = 11.416 (2) Å
α = 102.76 (3)°
β = 94.23 (3)°
γ = 109.75 (3)°
*V* = 584.0 (2) Å^3^
*Z* = 2
Mo *K*α radiation
μ = 0.27 mm^−1^
*T* = 153 (2) K
0.30 × 0.24 × 0.18 mm

#### Data collection

Rigaku R-AXIS RAPID IP area-detector diffractometer
Absorption correction: multi-scan (*ABSCOR*; Higashi, 1995 ▶) *T*_min_ = 0.924, *T*_max_ = 0.953
4589 measured reflections
2061 independent reflections
1712 reflections with *I* > 2σ(*I*)
*R*_int_ = 0.017

#### Refinement

*R*[*F* ^2^ > 2σ(*F* ^2^)] = 0.037
*wR*(*F* ^2^) = 0.109
*S* = 1.08
2061 reflections
146 parameters
H-atom parameters constrained
Δρ_max_ = 0.23 e Å^−3^
Δρ_min_ = −0.27 e Å^−3^

### 

Data collection: *RAPID-AUTO* (Rigaku, 2004 ▶); cell refinement: *RAPID-AUTO*; data reduction: *RAPID-AUTO*; program(s) used to solve structure: *SHELXTL* (Sheldrick, 2008 ▶); program(s) used to refine structure: *SHELXTL*; molecular graphics: *SHELXTL*; software used to prepare material for publication: *SHELXTL*.

## Supplementary Material

Crystal structure: contains datablocks I, global. DOI: 10.1107/S1600536808007733/cv2391sup1.cif

Structure factors: contains datablocks I. DOI: 10.1107/S1600536808007733/cv2391Isup2.hkl

Additional supplementary materials:  crystallographic information; 3D view; checkCIF report

## Figures and Tables

**Table 1:** Hydrogen-bond geometry (Å, °)

*D*—H⋯*A*	*D*—H	H⋯*A*	*D*⋯*A*	*D*—H⋯*A*
C2—H2*B*⋯O3^ii^	0.95	2.35	3.279 (2)	165

Symmetry code: (ii) 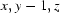.

**Fig. 1.**
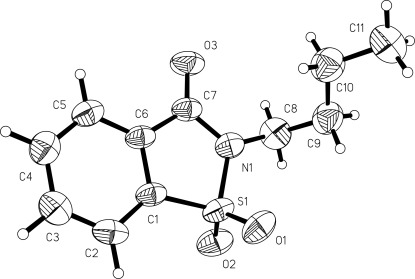
The molecular structure of (I) showing the atomic numbering and displacement ellipsoids drawn at the 40% probability level.

## supplementary crystallographic information

### Comment

The title compound, (I), also called THIAZONE, is a new skin penetration enhancer. The tests of penetration enhancing behaviors to berberine, ciclopirox olamino and cypermethrin show that penetration enhancing effect ofTHIAZONE is 2.99 times higher than that of AZONE. THIAZONE is widely applied in pharmaceutic industry, cosmetic and health care industry, agriculture and forest industry, and many others (Xiong, 2004). Herewith we report the crystal structure of (I).

In (I) (Fig. 1), all bond lengths and angles within the saccharin group are similar to those observed in the series of N-saccharin acids (Feeder & Jones, 1996), N-saccharin peracids (Feeder & Jones, 1994) and saccharin (Glidewell *et al.*, 2000).

In the crystal, the relatively short distance between the centroids of benzene rings from neighbouring molecules (Table 1) suggests an existence of π···π interactions. The crystal packing exhibits also exhibits weak intermolecular C—H···O hydrogen bonds (Table 2), which link the molecules related by translation along *b* axis into chains.

### Experimental

The title compound has been synthesized following the known procedure (Rice & Pettit, 1954). Saccharin sodium 2.65 g (0.011 mol) was dissolved in 20 ml of dried DMF. To the solution,1-butyl bromide 1.37 g (0.01 mol) was added. The mixture was stirred for half an hour at room temperature and then the mixture was heated with strring for 2 h at 100° C. The mixture was poured into water, and 2.50 g of the product were obtained (yield 95.7%). Single crystals suitable for X-ray measurement were obtained by recrystallization from dichloromethane at room temperature.

### Refinement

All H atoms were placed in idealized positions and constrained to ride on their parent atoms,with C—H distances of 0.95 (aromatic), 0.98 (CH_3_) and 0.99 Å (CH_2_),and with *U*_iso_(H) values set at 1.5 *U*_eq_(C)(for CH_3_) or 1.2 *U*_eq_(C)(for CH_2_, aromatic CH).

### Crystal data

**Table:**

C_11_H_13_NO_3_S	*Z* = 2
*M**_r_* = 239.28	*F*(000) = 252
Triclinic, *P*1	*D*_x_ = 1.361 Mg m^−^^3^
Hall symbol: -P 1	Mo *K*α radiation, λ = 0.71073 Å
*a* = 7.3130 (15) Å	Cell parameters from 1494 reflections
*b* = 7.7219 (15) Å	θ = 2.6–26.4°
*c* = 11.416 (2) Å	µ = 0.27 mm^−^^1^
α = 102.76 (3)°	*T* = 153 K
β = 94.23 (3)°	Block, colourless
γ = 109.75 (3)°	0.30 × 0.24 × 0.18 mm
*V* = 584.0 (2) Å^3^	

### Data collection

**Table:**

Rigaku R-Axis Rapid IP area-detector diffractometer	2061 independent reflections
Radiation source: Rotating Anode	1712 reflections with *I* > 2σ(*I*)
graphite	*R*_int_ = 0.017
ω Oscillation scans	θ_max_ = 25.0°, θ_min_ = 3.0°
Absorption correction: multi-scan (*ABSCOR*; Higashi, 1995)	*h* = −8→8
*T*_min_ = 0.924, *T*_max_ = 0.953	*k* = −9→9
4589 measured reflections	*l* = −13→13

### Refinement

**Table:**

Refinement on *F*^2^	Secondary atom site location: difference Fourier map
Least-squares matrix: full	Hydrogen site location: inferred from neighbouring sites
*R*[*F*^2^ > 2σ(*F*^2^)] = 0.037	H-atom parameters constrained
*wR*(*F*^2^) = 0.109	*w* = 1/[σ^2^(*F*_o_^2^) + (0.0554*P*)^2^ + 0.1349*P*] where *P* = (*F*_o_^2^ + 2*F*_c_^2^)/3
*S* = 1.08	(Δ/σ)_max_ = 0.001
2061 reflections	Δρ_max_ = 0.23 e Å^−^^3^
146 parameters	Δρ_min_ = −0.27 e Å^−^^3^
0 restraints	Extinction correction: *SHELXL*, Fc^*^=kFc[1+0.001xFc^2^λ^3^/sin(2θ)]^-1/4^
Primary atom site location: structure-invariant direct methods	Extinction coefficient: 0.129 (10)

### Special details

**Table:**

Geometry. All e.s.d.'s (except the e.s.d. in the dihedral angle between two l.s. planes) are estimated using the full covariance matrix. The cell e.s.d.'s are taken into account individually in the estimation of e.s.d.'s in distances, angles and torsion angles; correlations between e.s.d.'s in cell parameters are only used when they are defined by crystal symmetry. An approximate (isotropic) treatment of cell e.s.d.'s is used for estimating e.s.d.'s involving l.s. planes.
Refinement. Refinement of *F*^2^ against ALL reflections. The weighted *R*-factor *wR* and goodness of fit *S* are based on *F*^2^, conventional *R*-factors *R* are based on *F*, with *F* set to zero for negative *F*^2^. The threshold expression of *F*^2^ > σ(*F*^2^) is used only for calculating *R*-factors(gt) *etc*. and is not relevant to the choice of reflections for refinement. *R*-factors based on *F*^2^ are statistically about twice as large as those based on *F*, and *R*- factors based on ALL data will be even larger.

### Fractional atomic coordinates and isotropic or equivalent isotropic displacement parameters (Å^2^)

**Table:**

	*x*	*y*	*z*	*U*_iso_*/*U*_eq_	
S1	0.42409 (7)	0.81664 (7)	0.72568 (5)	0.0606 (2)	
O1	0.3065 (2)	0.7267 (2)	0.80490 (15)	0.0795 (5)	
O2	0.6279 (2)	0.8432 (2)	0.74251 (17)	0.0828 (5)	
O3	0.3009 (2)	1.1828 (2)	0.60625 (15)	0.0757 (5)	
N1	0.4016 (2)	1.0256 (2)	0.73053 (15)	0.0597 (4)	
C1	0.3124 (3)	0.7195 (3)	0.57339 (18)	0.0521 (5)	
C2	0.2711 (3)	0.5368 (3)	0.5017 (2)	0.0624 (5)	
H2B	0.3056	0.4455	0.5327	0.075*	
C3	0.1780 (3)	0.4928 (3)	0.3836 (2)	0.0685 (6)	
H3A	0.1489	0.3691	0.3317	0.082*	
C4	0.1262 (3)	0.6256 (3)	0.3393 (2)	0.0675 (6)	
H4A	0.0605	0.5910	0.2580	0.081*	
C5	0.1682 (3)	0.8068 (3)	0.41115 (19)	0.0603 (5)	
H5A	0.1332	0.8977	0.3800	0.072*	
C6	0.2625 (3)	0.8541 (3)	0.52964 (18)	0.0506 (4)	
C7	0.3201 (3)	1.0391 (3)	0.62121 (19)	0.0555 (5)	
C8	0.4943 (3)	1.1887 (3)	0.8376 (2)	0.0770 (7)	
H8A	0.5302	1.3073	0.8103	0.092*	
H8B	0.6178	1.1805	0.8731	0.092*	
C9	0.3700 (4)	1.2038 (4)	0.9357 (2)	0.0828 (7)	
H9A	0.3341	1.0851	0.9628	0.099*	
H9B	0.4509	1.3101	1.0061	0.099*	
C10	0.1870 (4)	1.2355 (4)	0.9009 (3)	0.0896 (8)	
H10A	0.0964	1.1205	0.8392	0.108*	
H10B	0.2196	1.3434	0.8629	0.108*	
C11	0.0818 (5)	1.2781 (4)	1.0081 (3)	0.1019 (9)	
H11A	−0.0369	1.2987	0.9794	0.153*	
H11B	0.1698	1.3930	1.0690	0.153*	
H11C	0.0449	1.1701	1.0446	0.153*	

### Atomic displacement parameters (Å^2^)

**Table:**

	*U*^11^	*U*^22^	*U*^33^	*U*^12^	*U*^13^	*U*^23^
S1	0.0564 (3)	0.0666 (4)	0.0714 (4)	0.0257 (3)	0.0102 (2)	0.0377 (3)
O1	0.0874 (11)	0.0866 (11)	0.0758 (10)	0.0256 (9)	0.0202 (8)	0.0509 (9)
O2	0.0591 (9)	0.0988 (12)	0.1038 (12)	0.0374 (8)	0.0022 (8)	0.0426 (10)
O3	0.0906 (11)	0.0589 (9)	0.0971 (12)	0.0388 (8)	0.0214 (9)	0.0387 (8)
N1	0.0593 (10)	0.0567 (10)	0.0665 (10)	0.0210 (8)	0.0087 (8)	0.0232 (8)
C1	0.0454 (10)	0.0560 (11)	0.0688 (12)	0.0231 (8)	0.0181 (8)	0.0338 (9)
C2	0.0565 (11)	0.0555 (11)	0.0927 (16)	0.0284 (9)	0.0287 (11)	0.0358 (11)
C3	0.0599 (13)	0.0654 (13)	0.0783 (15)	0.0195 (10)	0.0227 (11)	0.0166 (11)
C4	0.0589 (12)	0.0796 (15)	0.0638 (13)	0.0218 (11)	0.0142 (10)	0.0228 (11)
C5	0.0533 (11)	0.0721 (13)	0.0695 (13)	0.0275 (10)	0.0156 (9)	0.0368 (11)
C6	0.0439 (9)	0.0530 (10)	0.0669 (11)	0.0209 (8)	0.0164 (8)	0.0319 (9)
C7	0.0512 (11)	0.0544 (11)	0.0736 (13)	0.0235 (9)	0.0196 (9)	0.0322 (9)
C8	0.0637 (14)	0.0725 (15)	0.0824 (16)	0.0144 (11)	0.0050 (12)	0.0142 (12)
C9	0.0883 (17)	0.0833 (16)	0.0685 (14)	0.0276 (14)	−0.0029 (12)	0.0141 (12)
C10	0.0831 (17)	0.0983 (19)	0.0853 (17)	0.0308 (15)	0.0070 (14)	0.0255 (14)
C11	0.104 (2)	0.095 (2)	0.107 (2)	0.0410 (17)	0.0282 (18)	0.0150 (16)

### Geometric parameters (Å, °)

**Table:**

S1—O1	1.4243 (15)	C5—C6	1.383 (3)
S1—O2	1.4265 (16)	C5—H5A	0.9500
S1—N1	1.6661 (17)	C6—C7	1.476 (3)
S1—C1	1.747 (2)	C8—C9	1.503 (3)
O3—C7	1.210 (2)	C8—H8A	0.9900
N1—C7	1.383 (3)	C8—H8B	0.9900
N1—C8	1.470 (3)	C9—C10	1.481 (4)
C1—C2	1.384 (3)	C9—H9A	0.9900
C1—C6	1.386 (2)	C9—H9B	0.9900
C2—C3	1.381 (3)	C10—C11	1.527 (4)
C2—H2B	0.9500	C10—H10A	0.9900
C3—C4	1.383 (3)	C10—H10B	0.9900
C3—H3A	0.9500	C11—H11A	0.9800
C4—C5	1.375 (3)	C11—H11B	0.9800
C4—H4A	0.9500	C11—H11C	0.9800
			
Cg1···Cg1^i^	3.778 (2)		
			
O1—S1—O2	117.55 (10)	O3—C7—N1	123.7 (2)
O1—S1—N1	109.67 (10)	O3—C7—C6	126.81 (19)
O2—S1—N1	109.17 (10)	N1—C7—C6	109.45 (16)
O1—S1—C1	111.98 (10)	N1—C8—C9	115.25 (19)
O2—S1—C1	112.60 (10)	N1—C8—H8A	108.5
N1—S1—C1	93.09 (9)	C9—C8—H8A	108.5
C7—N1—C8	123.88 (18)	N1—C8—H8B	108.5
C7—N1—S1	114.58 (14)	C9—C8—H8B	108.5
C8—N1—S1	120.69 (15)	H8A—C8—H8B	107.5
C2—C1—C6	121.99 (19)	C10—C9—C8	115.6 (2)
C2—C1—S1	128.18 (16)	C10—C9—H9A	108.4
C6—C1—S1	109.81 (15)	C8—C9—H9A	108.4
C3—C2—C1	117.30 (19)	C10—C9—H9B	108.4
C3—C2—H2B	121.3	C8—C9—H9B	108.4
C1—C2—H2B	121.3	H9A—C9—H9B	107.4
C2—C3—C4	121.1 (2)	C9—C10—C11	113.4 (2)
C2—C3—H3A	119.4	C9—C10—H10A	108.9
C4—C3—H3A	119.4	C11—C10—H10A	108.9
C5—C4—C3	121.1 (2)	C9—C10—H10B	108.9
C5—C4—H4A	119.4	C11—C10—H10B	108.9
C3—C4—H4A	119.4	H10A—C10—H10B	107.7
C4—C5—C6	118.64 (19)	C10—C11—H11A	109.5
C4—C5—H5A	120.7	C10—C11—H11B	109.5
C6—C5—H5A	120.7	H11A—C11—H11B	109.5
C5—C6—C1	119.82 (19)	C10—C11—H11C	109.5
C5—C6—C7	127.23 (17)	H11A—C11—H11C	109.5
C1—C6—C7	112.96 (17)	H11B—C11—H11C	109.5
			
O1—S1—N1—C7	−117.32 (15)	C4—C5—C6—C7	−179.82 (17)
O2—S1—N1—C7	112.58 (16)	C2—C1—C6—C5	0.2 (3)
C1—S1—N1—C7	−2.65 (15)	S1—C1—C6—C5	−178.69 (14)
O1—S1—N1—C8	72.84 (17)	C2—C1—C6—C7	179.99 (16)
O2—S1—N1—C8	−57.26 (18)	S1—C1—C6—C7	1.14 (19)
C1—S1—N1—C8	−172.49 (16)	C8—N1—C7—O3	−7.0 (3)
O1—S1—C1—C2	−65.33 (19)	S1—N1—C7—O3	−176.51 (15)
O2—S1—C1—C2	69.76 (19)	C8—N1—C7—C6	173.10 (17)
N1—S1—C1—C2	−177.99 (17)	S1—N1—C7—C6	3.63 (19)
O1—S1—C1—C6	113.43 (14)	C5—C6—C7—O3	−3.0 (3)
O2—S1—C1—C6	−111.48 (14)	C1—C6—C7—O3	177.15 (18)
N1—S1—C1—C6	0.77 (14)	C5—C6—C7—N1	176.82 (17)
C6—C1—C2—C3	0.2 (3)	C1—C6—C7—N1	−3.0 (2)
S1—C1—C2—C3	178.82 (14)	C7—N1—C8—C9	101.9 (2)
C1—C2—C3—C4	−0.7 (3)	S1—N1—C8—C9	−89.2 (2)
C2—C3—C4—C5	0.9 (3)	N1—C8—C9—C10	−63.7 (3)
C3—C4—C5—C6	−0.5 (3)	C8—C9—C10—C11	−171.7 (2)
C4—C5—C6—C1	0.0 (3)		

Symmetry codes: (i) −*x*+2, −*y*+1, −*z*+1.

### Hydrogen-bond geometry (Å, °)

**Table:**

*D*—H···*A*	*D*—H	H···*A*	*D*···*A*	*D*—H···*A*
C2—H2B···O3^ii^	0.95	2.35	3.279 (2)	165.

Symmetry codes: (ii) *x*, *y*−1, *z*.
